# Cell-to-cell communication in cancer: workshop report

**DOI:** 10.1038/npjbcancer.2015.22

**Published:** 2015-11-25

**Authors:** Maja H Oktay, Yi-Fen Lee, Allison Harney, Dorothy Farrell, Nastaran Z Kuhn, Stephanie A Morris, Emily Greenspan, Suresh Mohla, Piotr Grodzinski, Larry Norton

**Affiliations:** 1 Department of Pathology, Montefiore Medical Center, New York, NY, USA; 2 Department of Pathology, University of Rochester Medical Center, Rochester, MN, USA; 3 Department of Anatomy & Structural Biology, Albert Einstein College of Medicine, New York, NY, USA; 4 Office of Cancer Nanotechnology Research, National Cancer Institute, Bethesda, MD, USA; 5 Division of Cancer Biology, National Cancer Institute, Rockville, MD, USA; 6 Center for Strategic Scientific Initiatives, National Cancer Institute, Bethesda, MD, USA; 7 Department of Medicine, Memorial Sloan Kettering Cancer Center, New York, NY, USA; 8 The Breast Cancer Research Foundation, New York, NY, USA

## Abstract

Recent advances in cancer biology and the development of new research tools have enabled interrogations of single cells and cell–cell interactions. Emerging technologies are capable of revealing data on the physical characteristics of cells, differences in the genome and proteome between cancerous and healthy cells, and variations in distinct cell subpopulations. Dynamic measurements enable studies that can reveal the evolution of cell characteristics. Cells can also be assembled *in vitro* or *ex vivo* into two- and three-dimensional cell environments, allowing for studies of cell–cell interactions and cell signaling. The Memorial Sloan Kettering Cancer Center, in collaboration with the Breast Cancer Research Foundation and the National Cancer Institute, co-organized a workshop as an opportunity for leading researchers in their respective fields to present and discuss scientific research highlights relevant to the utilization of techniques and technologies for studying cell-to-cell communications in cancer. Avenues of future development and the potential for clinical utility were primary features of these discussions. The scientific presentations and extensive ensuing discussions resulted in the identification of a number of research opportunities, which are summarized in this report.

## Introduction

Advanced technologies such as nanotechnologies and microfluidics platforms as well as conventional three-dimensional (3D) organotypic cultures have contributed to an increased understanding of cancer biology at the single-cell level and at the level of communication between cells. This in turn is leading to a greater appreciation of the implications of intratumoral heterogeneity and the role of tumor microenvironment and vasculature in cancer progression and metastasis. Both, the Breast Cancer Research Foundation (BCRF) and the National Cancer Institute (NCI) support research in these areas. In collaboration with the Memorial Sloan Kettering Cancer Center (MSKCC), the BCRF and NCI convened a workshop titled “Cell-to-Cell Communication in Cancer” that was hosted by MSKCC on 14–15 July 2014. The workshop consisted of plenary presentations from researchers working at the interface of biology and engineering interspersed with breakout sessions to discuss aspects of cancer biology for which cellular analyses can yield clinically relevant insights into tumor behavior. Breakout session discussions focused on intratumoral heterogeneity, single-cell measurements, and modeling complex tumor microenvironment interactions. Discussions were aimed at identifying ways in which the characterization of single cells and cell-to-cell interactions could improve the understanding and the treatment of cancer.

A number of themes emerged from the presentations and discussions, including the diversity of factors that contribute to malignancy, heterogeneity, and the evolution of progression-linked mutations; the importance of intercellular signaling in tumor progression and invasion; and the need to develop *in vitro* and *ex vivo* models that capture these factors and can predict tumor response to intervention. This report highlights the presentations and findings made by participants in three key areas—improving cancer diagnosis and therapy using single-cell analyses; the effects of cell-to-cell communication within the tumor microenvironment on cancer progression and metastasis; and methods and models for recapitulating the tumor microenvironment.

## Single-cell analyses

Recent technological advances have enabled the detailed genetic and proteomic analysis of single tumor cells, as well as the isolation and analysis of either intact circulating tumor cells (CTCs) or circulating DNA from the blood of cancer patients.^1^ These techniques have already demonstrated clinical utility, such as use of CTCs as prognostic markers for advanced prostate cancer.^2^ Workshop presentations focused on the ways in which these technologies have also enabled fundamental studies of cancer cell signaling pathways,^3^ genomic heterogeneity and evolution,^4,5^ and physical characteristics.^6,7^ Both presentations and discussions highlighted the potential for insights from these studies to spark discovery and development of new therapeutic strategies and associated diagnostics.

Howard Scher discussed the use of CTC analysis to provide non-invasive patient monitoring, including the possibility of capturing the transformation into therapy resistant disease in prostate cancer by tracking androgen receptor status of CTCs. Scher views CTC analysis as more effective at capturing the heterogeneity of cancer cells than traditional bulk analysis of the tumor, and therefore potentially more likely to discover drug resistance as it emerges. The evolution of disease and emergence of therapy-resistant phenotypes has important implications for cancer treatment, and Nicholas Navin presented his work revealing that, in at least some cancers, evolution occurs not gradually but rather by stepwise clonal expansions, with different mutational types (e.g., copy number rearrangements versus point mutations) having quite different natural histories. Wei Wei focused his talk on analyzing intracellular signaling pathways in single tumor cells and how integrated genomics and functional proteomics studies on single tumor cells reveal connections between genomic information and biological function. By analyzing protein networks associated with acquired drug resistance in glioblastoma multiforme, his team is developing strategies to predict and pre-empt resistance through the use of targeted combination therapies. Scott Manalis demonstrated the use of his suspended microchannel resonator device to measure the mass of single cells in real time and showed that growth measurements on genetically characterized single cells can reveal the correlation between treatment efficacy and genomic features in a way that cannot be replicated in bulk tumor studies.

These presentations provided rich data and context for discussions centered on exploiting single-cell analyses to improve cancer diagnosis and therapy. Participants discussed immediate opportunities to use single-cell studies, particularly involving CTCs, to monitor drug response and to identify and target the most aggressive phenotypes within a tumor. At the same time, participants recognized that certain important aspects of cell or tumor behavior cannot be deduced at single-cell level measurements, and for many single-cell assays there is a need to establish the number of measurements allowing for reliable predictions. In addition, there is currently little consensus on what would be the most useful functional assays (e.g., viability, immune/cytokine assays) to characterize tumor cells or CTCs, especially from solid tumors.

Discussions led to the identification of additional questions that must be addressed before the results of these analyses could be translated into clinical strategies. For example, what is the role of intratumoral heterogeneity in treatment response and what is the clinical utility of measuring this diversity at the single cell level? These questions immediately raise the issue of resistance-linked mutations, and whether they are pre-existing, or a result of therapy(ies) that may induce or select for specific mutations, or both. Greater understanding of how surviving cancer cells adapt to therapy and the post-therapy environment is necessary to enable targeting of these cells without promoting more resistant or aggressive disease. All of these considerations indicate that the disease state must be analyzed over time, potentially using mutations as markers of lineage evolution. Practical limitations on biopsy collection for many cancer types suggest CTCs are one of the only avenues through which these longitudinal data can be collected.

Several participants emphasized that the focus on genomic and functional analyses should not lead to neglect of the physical parameters and phenotypes of cells, which may not only indicate the level of heterogeneity and malignancy but may also influence them. For example, it is not known how physical constraints such as high pressure within tumors, and the eventual release from these constraints through extravasation or migration, affect the invasiveness or metastatic potential of cancer cells. Another important open question in cellular characterization is how to distinguish dormant cancer cells from normal cells, and then differentially target the dormant cells. This targeting will require determining the relative importance of cell-intrinsic and cell-extrinsic influences in dormancy.

## Tumor microenvironment

The importance of the tumor microenvironment was understood from the outset of the workshop, which began with a plenary address by Mina Bissell on the effect of tissue architecture on gene expression and subsequently cell behavior. Bissell’s group discovery that oncogenes induce the malignant phenotype only in the proper context of the microenvironment^8^ began a 30-year program on the study of tissue architecture and its effect on malignancy. Bissell’s work shows that the profound effect microenvironment can have on cell phenotype, even dominating over the cellular genotype,^9^ and the importance of 3D models that recapitulate the tumor microenvironment and cellular interactions to properly understand the sequence of progression. In 3D gel culture systems, phenotypic characteristics of malignant transformation that are often considered to be a consequence of alterations in the genome have the ability to activate oncogenic signaling pathways and contribute to malignant transformation. For example, increased glycolysis in a 3D model can become an oncogenic event,^10^ and dormant cells residing in metastatic microvascular niches could be reactivated upon loss of endothelial-derived thrombospondin-1 that coincide with the onset of endothelial sprouting.^11^ Other findings with particularly strong clinical relevance are that cell attachment to basement membrane and formation of polarized tridimensional structures protect from chemotherapy-induced apoptosis,^12^ and manipulation of integrin signaling in 3D matrices can increase cancer-cell sensitivity to ionizing radiation.^13^

Echoing Bissell’s findings on the importance of paracrine signaling in malignancy, Clifford Hudis spoke on the relationship between obesity, inflammation, and cancer. He presented a series of analyses on adipose tissue from different malignancies and found “crown-like structures” present in the white adipose tissue, which correlate positively with excess weight, serum levels of inflammatory cytokines, aromatase, and worse outcomes in some cancers. Paul Newton and Johanna Joyce both discussed the role of cellular communication in breast cancer metastasis. Newton employs evolutionary game theory models of single-cell and cell population trafficking to study cancer progression from one site to another.^14,15^ Using a breast cancer patient data set, including outcomes and ER/Her2 status, possible anatomic pathways and progression timescales of metastases were compared and probability distributions of cell migration patterns were established. Joyce’s work focuses on understanding the mechanisms of tumor metastasis by investigating how the microenvironment responds to metastatic tumor cells and how heterotypic interactions between tumor and stromal cells differ between secondary organ metastasis sites.^16^ By comparing gene expression array profiles from tumor and stroma cells in micro- and macromestases in the brain, lung, and bone, they identified a protease, cathepsin S, with high specificity for brain metastases. These studies indicate that cellular signaling and trafficking patterns can provide both diagnostic signatures and therapeutic targets for metastasis.

Each of these presentations highlighted scenarios in which studies of cancer cells in context are necessary to predict or understand how the phenotype, or even genotype, of those cells will progress. Workshop participants recognized numerous implications of these findings, which point to the continued relevance of cancer-cell phenotype even in the era of genomic medicine. Participants emphasized that for clinicians, the questions of which phenotypes to target and how to identify them will be crucial to determining treatment strategies. Clinicians also need to be able to identify and target dormant cells and understand catastrophic events that lead to pan resistance in metastatic cells and rapid patient death.

Better understanding of the activation of dormant cells and what triggers the metastatic pathway in these cells is necessary to identify targets to short circuit this pathway and improve outcomes in cancer care. The role of inflammation in activating dormant cells, along with the evidence of collective behavior underlying progression and metastasis, point to the need to study cell-to-cell communication within the tumor microenvironment. Participants were excited at the prospect of looking to the tumor stroma to provide clues to which lesions are likely to progress over time, potentially providing a route to limit overdiagnosis and overtreatment of indolent tumors, which result in few saved lives but significant health care and quality of life costs. Participants also raised the prospect of identifying another phenotypic target for intervention besides mitosis, perhaps a migratory, dissemination-linked, or inflammatory signature.

In thinking about CTCs in the context of the tumor microenvironment, participants raised the idea of CTC “memory”, or the ability of a CTC to survive outside the context of the primary tumor and its microenvironment. It was speculated that perhaps those CTC subtypes that are associated with metastasis are those that retain some “memory” of the primary tumor’s microenvironment across an as yet to be determined timescale. Answering this question depends on understanding several other not well-defined processes, such as identifying the initiating event in a cell becoming a CTC and determining what drives molecular alterations, such as gene mutations, during circulation. In any case, it was deemed important to define the differences between single-cell assays in the context of the primary tumor versus CTC assays where tumor cells survive without the tumor microenvironment.

## 3D tumor models

The final session of the workshop focused on the development of accurate 3D *in vitro* or *ex vivo* models of tissues that mimic the physiological conditions in the tumor microenvironment and recapitulate individual aspects in the metastatic cascade. David Beebe is developing microfluidic tools that balance the ability to isolate specific interactions between cells *in vitro* and the more realistic complexity but greater cost of *in vivo* models. His group has used a microscale cell-culture platform capable of compartmentalizing adherent and non-adherent cells in controlled environments to understand and predict patient response to the proteasome inhibitor bortezomib.^17^ Only in cultures with both multiple myeloma and stromal cells were tumor cell viabilities similar to patient response to bortezomib, indicating that this platform can be used to recapitulate clinical response *in vitro*.

Christopher Chen described the use of 3D printed networks to recreate vascular networks for the study of angiogenesis.^18^ Endothelial cells are seeded inside a network that mimics *in vivo* blood vessels, and via a parallel channel, angiogenic factors can be flowed through to identify combinations that produce different types of endothelial cell invasion.^19^ The system allows for the visualization of sprouting angiogenesis over time, providing mechanistic information on the function of different growth factors and the mechanism underlying treatment with anti-angiogenic drugs. Roger Kamm described his efforts to understand tumor-cell migration through reconstruction of blood flow in a microfluidic device to study intravasation and extravasation. Intravasation increased in the presence of macrophages, and macrophage-induced intravasation is associated with increased endothelial permeability, which can be stimulated by the addition of tumor necrosis factor-α.^20^ These studies indicate the role of non-neoplastic cells and signaling in migration and metastasis and demonstrate that 3D models can illuminate the processes underlying these events, as well as malignant transformation and progression.

Workshop participants discussed the minimum requirements of the multiple available *in vitro* or *ex vivo* models to be physiologically relevant to the *in vivo* environment. Although there was consensus that the complexity of the model depends on the experimental question(s), it was agreed that issues of vascularization and the viability of endothelial cells in mixed cultures and organoids, as well as how to retain viability of stromal cells, are inescapable. Capturing the clinically relevant characteristics of the tumor microenvironment, such as oxygen levels, hypoxia, and necrosis, will probably require use of patient-derived models or tissue fragments, rather than dissociated cells, and multiple models may be necessary to study different aspects of tumor microenvironment interactions. The important factor will be that a model captures the relevant cell types, tissue structure, and cancer molecular subtypes being probed in a given investigation.

Participants did recognize that coupling technologies to create a master model in which multiple aspects of a tumor (physical, chemical, and biological) are simultaneously interrogated and multiple discrete steps in tumor progression are recapitulated would enable major strides in understanding cancer-cell behavior. There was consensus on the importance of using “humanized” *in vitro*/*ex vivo* models, and that these models should incorporate information derived from other model systems to improve performance. Validation in human samples to ensure relevance to human disease was also called for during the discussions. In the development of models, in particular, the group called for team science approaches, with strong engagement of clinicians, clinical researchers, basic cancer biologists, and engineers.

## Opportunities, barriers, and solutions

Workshop participants identified numerous opportunities for both discovery oriented research and clinical applications based on technologies that characterize single cells or enable study of cell-to-cell communications. New technologies are being used to capture and analyze tumor heterogeneity, and the associated implications for patient outcomes. Further studies in this area are expected to have major impact on both cancer biology and patient care through improved choice of therapeutic course, including more informed tumor surveillance. The identification of signatures of dormancy and reactivation in cancer cells is also expected to be clinically actionable. Integrated models of tumors that enable studies of cellular behavior at multiple scales, and that can include or isolate environmental factors, will provide insight into cancer progression and disease markers that are badly needed.

Among the most clinically relevant technologies are those that can be used for in depth, longitudinal profiling of therapeutic response, such as CTC capture and analysis devices, and integrated approaches to diagnostics and therapy. Effective use of these techniques to characterize tumors and guide clinical decision making will require extensive studies to determine the correspondence between single-cell measurement results and bulk tumor characteristics. Patient-specific models to predict therapeutic response and rationalize treatment choices also have the potentially-to-radically alter treatment outcomes. Deployment of such models depends on further development of fully humanized *in vitro* and *ex vivo* models, which workshop participants saw as a pressing but not fully recognized need.

A central point of discussion among workshop participants was development of effective collaborations to capitalize on the strengths of researchers in each field. One item of concern is that many investigators are not aware of valuable experimental models and tools that are available and currently in use by other researchers. Another concern is the difficulty of bringing together researchers from different disciplines and disease focus areas. Joint workshops, special sessions at larger meetings and smaller dedicated, Gordon Research Conference-type meetings were all suggested to bridge these gaps. However, participants noted friction at the social interface between disciplines at both institutions and conferences, which can lead to ineffective and inefficient technology development. For example, clinicians are frequently disillusioned by seeing engineers present technologies, devices, and strategies that are not clinically relevant, leading to decreased engagement.

There was some concern that the NCI’s emphasis on the R01 single project mechanism may make it difficult to pursue the sort of multidisciplinary, integrated research presented at the workshop. Participants saw a role for the NCI in bringing different disciplines together and facilitating long-term collaborations between clinicians and engineers. Possibly NCI may also be able to help address issues with clinician availability—often interested clinicians cannot engage in this type of research due to time constraints placed by their institutions/hospitals. This is particularly pressing for the newest generation of physician scientists, who face low and decreasing funding rates for research. In general, it is acknowledged that clinicians have a much larger time pressure than basic or applied scientists, but this hurdle can be overcome with the correct organizational structure.
